# The secreted PAMP-induced peptide StPIP1_1 activates immune responses in potato

**DOI:** 10.1038/s41598-023-47648-x

**Published:** 2023-11-23

**Authors:** Linda Nietzschmann, Ulrike Smolka, Elvio Henrique Benatto Perino, Karin Gorzolka, Gina Stamm, Sylvestre Marillonnet, Katharina Bürstenbinder, Sabine Rosahl

**Affiliations:** 1 Department Biochemistry of Plant Interactions, Leibniz Institute of Plant Biochemistry, Weinberg 3, 06120 Halle (Saale), Germany; 2 Department Molecular Signal Processing, Leibniz Institute of Plant Biochemistry, Weinberg 3, 06120 Halle (Saale), Germany; 3 Department of Cell and Metabolic Biology, Leibniz Institute of Plant Biochemistry, Weinberg 3, 06120 Halle (Saale), Germany

**Keywords:** Biochemistry, Plant sciences

## Abstract

Treatment of potato plants with the pathogen-associated molecular pattern Pep-13 leads to the activation of more than 1200 genes. One of these, *StPIP1_1*, encodes a protein of 76 amino acids with sequence homology to PAMP-induced secreted peptides (PIPs) from *Arabidopsis thaliana*. Expression of *StPIP1_1* is also induced in response to infection with *Phytophthora infestans*, the causal agent of late blight disease. Apoplastic localization of StPIP1_1-mCherry fusion proteins is dependent on the presence of the predicted signal peptide. A synthetic peptide corresponding to the last 13 amino acids of StPIP1_1 elicits the expression of the *StPIP1_1* gene itself, as well as that of pathogenesis related genes. The oxidative burst induced by exogenously applied StPIP1_1 peptide in potato leaf disks is dependent on functional StSERK3A/B, suggesting that StPIP1_1 perception occurs via a receptor complex involving the co-receptor StSERK3A/B. Moreover, StPIP1_1 induces expression of *FRK1* in Arabidopsis in an RLK7-dependent manner. Expression of an RLK from potato with high sequence homology to AtRLK7 is induced by StPIP1_1, by Pep-13 and in response to infection with *P. infestans*. These observations are consistent with the hypothesis that, upon secretion, StPIP1_1 acts as an endogenous peptide required for amplification of the defense response.

## Introduction

To defend themselves against pathogens, plants recognize not only pathogen-associated molecular patterns (PAMPs), but also structures derived from damage that has occurred during infection. Similar to PAMPs, these damage-associated molecular patterns (DAMPs) bind to plasma membrane-localized receptor kinases, leading to the activation of immune responses. DAMPs can be released passively upon cell disruption, as is the case for cell wall fragments and extracellular plant-derived compounds such as ATP^1^, NAD(P)^2^ and DNA^3^.

In contrast, there are endogenous danger signals that are actively processed and released into the apoplastic space. These include peptides that are produced from preproproteins, such as systemin^4^, rapid alkalinization factor RALF^5^, plant elicitor peptides (PEPs^6^), as well as the serine rich endogenous peptides (SCOOP^7^) and PAMP-induced peptides, PIPs^8^. These peptides, termed phytocytokines^9–11^), are perceived by plasma membrane receptors^8, 12–14^, thus activating immune responses such as cytosolic Ca^2+^ elevation, reactive oxygen species (ROS) burst, MAP kinase activation and transcriptional reprogramming^8, 15, 16^.

The family of SGP-rich secreted endogenous peptides comprises not only the immunity-related PIPs and SCOOPs, but also Inflorescence Deficient in Abscission (IDA) peptides, which are involved in floral abscission^17^, thus emphasizing the dual role of phytocytokines in immunity and development. Apoplastic localization has been shown for Arabidopsis AtPIP1^8^, SCOOP10^18^ and IDA-like7 (IDL7^19^), where they are postulated to bind to their respective receptors RLK7^8^, MIK2^20^ and HAESA (HAE)/HAESA-LIKE2 (HSL2^17^).

In Arabidopsis, PIPs are encoded by 11 genes. Similar to PEP1^21^, AtPIP1 induces stomatal closure^22^, suggesting a role in stomatal immunity. Recently, the PAMP-induced peptides StPIP1 to StPIP4 were described in potato (*Solanum tuberosum*^23^). Although exogenous application of chemically synthesized StPIP1 to leaves does not induce increases in the cytosolic Ca^2+^ concentrations nor ROS production, transgenic potato plants overexpressing StPIP1 show enhanced defense gene expression upon virus infection, suggesting a role of StPIP in eliciting defense responses^23^.

One of the most devastating diseases of potato (*Solanum tuberosum*) is late blight, caused by the hemibiotrophic oomycete *Phytophthora infestans*. Treatment of susceptible plants with the PAMP Pep-13, a 13 amino acid oligopeptide from an extracellular transglutaminase from *Phytophthora* species, results in the activation of salicylic acid and jasmonic acid-dependent immune responses, such as an oxidative burst, defense gene activation and hypersensitive cell death^24–26^). Perception of Pep-13 requires the co-receptor BRI1-ASSOCIATED RECEPTOR KINASE1 (BAK1), encoded by two genes in potato, the SOMATIC EMBRYOGENESIS RECEPTOR KINASEs *StSERK3A* and *StSERK3B*^27^. Transgenic potato plants with down-regulated expression of *StSERK3A/B* are unable to mount an oxidative burst or to activate MAP kinases in response to Pep-13 treatment^27^.

We identified two novel *PIP1* genes in transcriptome analyses of Pep-13 activated genes. The two genes, *StPIP1_1* and *StPIP1_2*, encode the potato homologues of AtPIP1, whose secretion into the apoplast requires a signal peptide. Synthetic peptides encompassing the C-terminal 13 amino acids of StPIP1_1 induce an oxidative burst in a StSERK3A/B-dependent manner and activate defense gene expression in potato leaves.

## Results

### *StPIP1-1* and *StPIP1_2* are Pep-13-activated genes

**Figure 1:**
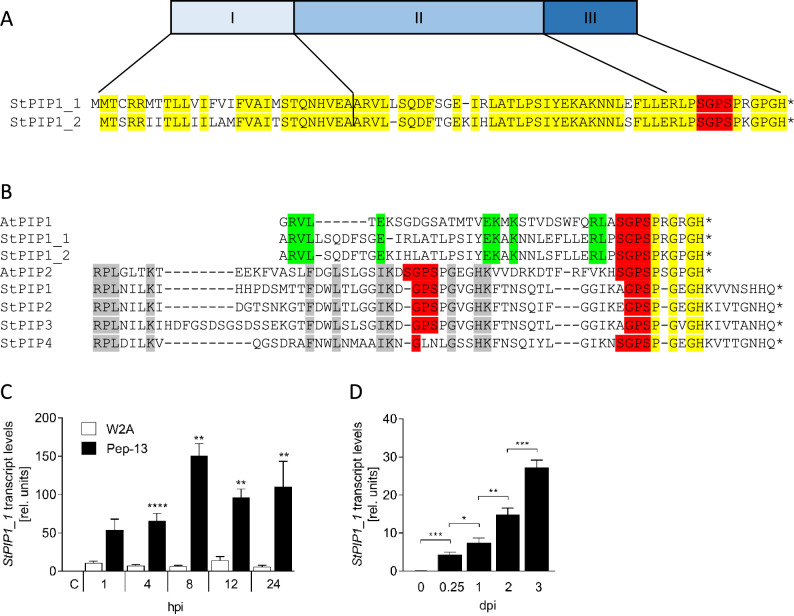
Structure of StPIP1_1 and expression in response to Pep-13 treatment and *P. infestans* infection. (**A**) Domain structure of StPIP1_1 and StPIP1-2. Domain I: signal peptide, domain II: variable region, domain III: conserved region. Conserved amino acids conserved are marked in yellow. The SGPS motif is marked in red. (**B**) Amino acid sequence of the predicted mature proteins AtPIP1 and AtPIP2 with those of potato StPIP1_1 and StPIP1_2, as well as StPIP1, 2, 3 and 4 (Combest et al.^23^). Amino acids conserved between AtPIP1 and StPIP1_1 and StPIP1_2 are marked in green, those conserved between AtPIP2 and StPIP1 to StPIP4 in grey and those conserved in all proteins in yellow. The SGPS and GPS motifs are marked in red. (**C**) *StPIP1_1* transcript accumulation in response to Pep-13. RNA was isolated from W2A (white bars) or Pep-13 (black bars) infiltrated potato leaves at the time points indicated (hours post infiltration, hpi) and subjected to qRT-PCR analyses. Data shown were normalized to the expression of *StEF1α* and are derived from three independent experiments (n ≥ 6, except for 1 hpi Pep-13: n = 5). Error bars represent SEM. Statistical differences between W2A and Pep-13-infiltrated plants were determined by Mann Whitney U test (**p ≤ 0.001; ****p ≤ 0.0001). (**D**) *StPIP1_1* transcript accumulation in response to *P. infestans* infection. RNA was isolated from *P. infestans*-inoculated potato leaves at the time points indicated (days post inoculation, dpi) and subjected to qRT-PCR analyses. Data shown were normalized to the expression of S*tEF1a* and are derived from three independent experiments (n = 8). Error bars represent SEM. Statistical analyses were performed by Mann Whitney U test (*p ≤ 0.05; **p ≤ 0.01; ***p ≤ 0.005.

*StPIP1_1* and *StPIP1_2* were identified in microarray analyses as two of more than 1200 Pep-13-activated genes^28, 29^. Specifically, transcript abundance of PGSC0003DMG400014649 (*StPIP1_1*) and PGSC0003DMG400006400 (*StPIP1_2*), predicted to encode 76 and 75 amino acid proteins, respectively, was enhanced in potato leaves after Pep-13 infiltration, compared to infiltration of the nearly inactive analogue W2A (Fig. S1A–C). In addition to the predicted signal peptide (LocTree3; Fig. 1A), the two encoded proteins contain a variable region and a highly conserved C-terminal region spanning 13 amino acids (Fig. 1A). Eleven of the C-terminal 13 amino acids of StPIP1_1 (RLPSGPSPRGPGH) are identical to those of AtPIP1 (RLASGPSPRGRGH), whereas 10 out of 13 are identical in StPIP1_2 (Fig. 1B, left panel). Sequence comparison also reveals that the recently identified StPIP1 to StPIP4^23^ have higher sequence homology to AtPIP2 (Fig. 1B, right panel). Due to the high sequence homology to AtPIP1, StPIP1_1 was analyzed further. To gain insight into the temporal patterns of *StPIP1_1* induction in response to Pep-13 infiltration, *StPIP1* transcript levels were analyzed by qRT-PCR in time-course experiments (Fig. 1C). Phytochamber-grown potato plants were infiltrated with Pep-13 or the inactive analog W2A^24^. Expression of *StPIP1_1* was significantly increased by Pep-13 treatment after 4 h and remained enhanced, compared to W2A treatment, until the end of the experiment at 24 h. Additional transcriptome analyses revealed that *StPIP1_1* also responded to infection by *P. infestans*, as did *StPIP1_2* and *StPIP1-4* (Fig. S2). Time-course experiments showed that *StPIP1_1* transcript levels were significantly increased 2 and 3 days after inoculation with *P. infestans* (Fig. 1D). Thus, both PAMP-treatment and infection by *P. infestans* induced *StPIP1_1* gene expression.

### StPIP1_1 activates immune responses

**Figure 2:**
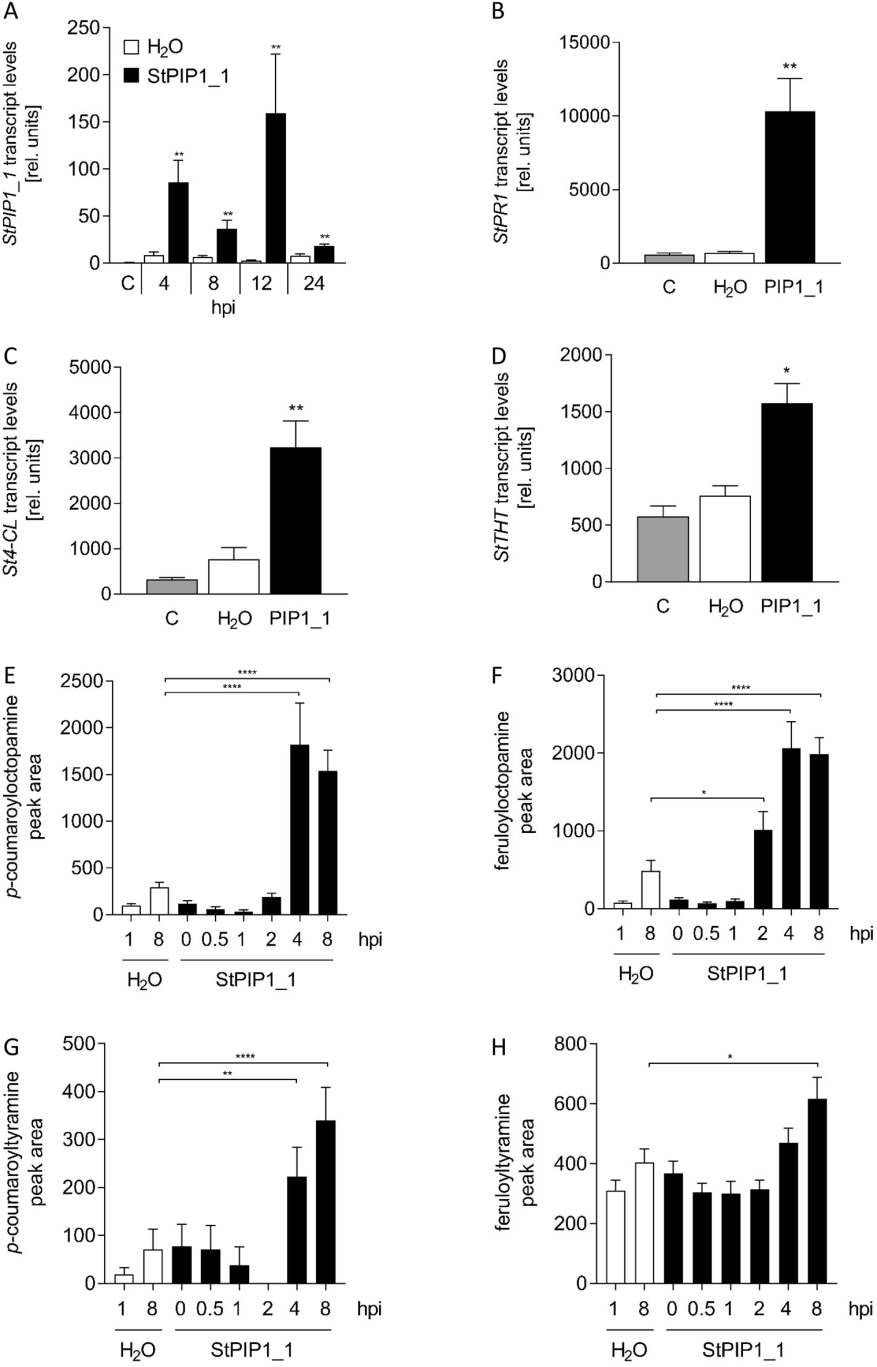
StPIP1_1 induces defense gene expression and accumulation of defense metabolites. (**A**–**D**) RNA was isolated from untreated (“C”, grey bar), water-treated (white bars) or StPIP1_1 (black bars) infiltrated potato leaves and subjected to qRT-PCR analyses. Data shown were normalized to the expression of *StEF1α* and are derived from two independent experiments. Error bars represent SEM. Statistical differences between water-treated and StPIP1_1-treated plants were determined by Mann Whitney U test (*p ≤ 0.05; **p ≤ 0.001). (**A**) *StPIP1_1* transcript levels after infiltration of StPIP1_1 at the time points indicated. n = 6. (**B**) *StPR1* transcript levels 4 h after infiltration of StPIP1_1. n ≥ 6. (**C**) *St4CL* transcript levels 4 h after infiltration of StPIP1_1. n = 5. (**D**) *StTHT* transcript levels 4 h after infiltration of StPIP1_1. n = 5. (**E**–**H**) Metabolite profiling was performed on methanolic extracts from potato leaves infiltrated with water or StPIP1_1 for the time points indicated (hours post infiltration). (**E**) *p*-coumaroyltyramine, (**F**) feruloyltyramine, (**G**) *p*-coumaroyloctopamine, (**H**) feruloyloctopamine. Data are derived from two independent experiments (n ≥ 17). Error bars represent SEM. Statistical differences were determined by Mann Whitney U test (*p ≤ 0.05; **p ≤ 0.01; ****p ≤ 0.001).

The C-terminal 13 amino acids of AtPIP1 were described to activate defense gene expression^8^. We therefore assessed the elicitor activity of a synthetic oligopeptide covering the respective C-terminal 13 amino acids of StPIP1_1 (Fig. 1B). Infiltration of this peptide into potato leaves resulted in enhanced *StPIP1_1* transcript levels starting 4 h after treatment (Fig. 2A). Moreover, expression of *StPR1* encoding the sterol binding protein *PATHOGENESIS-RELATED 1*^30^, *St4CL* (*4-COUMARATE-COA-LIGASE*) and *StTHT* (*TYRAMINE HYDROXYCINNAMOYL-COA TRANSFERASE*^31^) was enhanced in response to StPIP1_1 (Fig. 2B–D).

To test if altered defense-gene expression correlates with metabolic changes in StPIP1_1 treated plants, we determined the accumulation of specialized metabolites using untargeted metabolite profiling by UPLC-ESI-QTOF-MS of methanolic leaf extracts. Sixty-seven features were at least twofold higher in plants treated with StPIP1_1 for 8 h compared to water-treated plants or plants treated with StPIP1_1 for 1 h. In accordance with increased transcript levels of *4-CL* and *THT*, which encode enzymes involved in the synthesis of hydroxycinnamic acid amides, we identified the defense metabolites *p*-coumaroyl- and feruloyloctopamine (Fig. 2E,F) as well as *p*-coumaroyltyramine^32^ (Fig. 2G) as StPIP1_1-induced compounds. Feruloyltyramine, on the other hand, was less than twofold enhanced after 8 h (Fig. 2H). Thus, StPIP1_1 induced defense gene expression and accumulation of defense metabolites suggesting that perception of StPIP1_1 leads to the activation of several defense-related traits.

### StPIP1_1-mCherry is secreted into the apoplast

**Figure 3:**
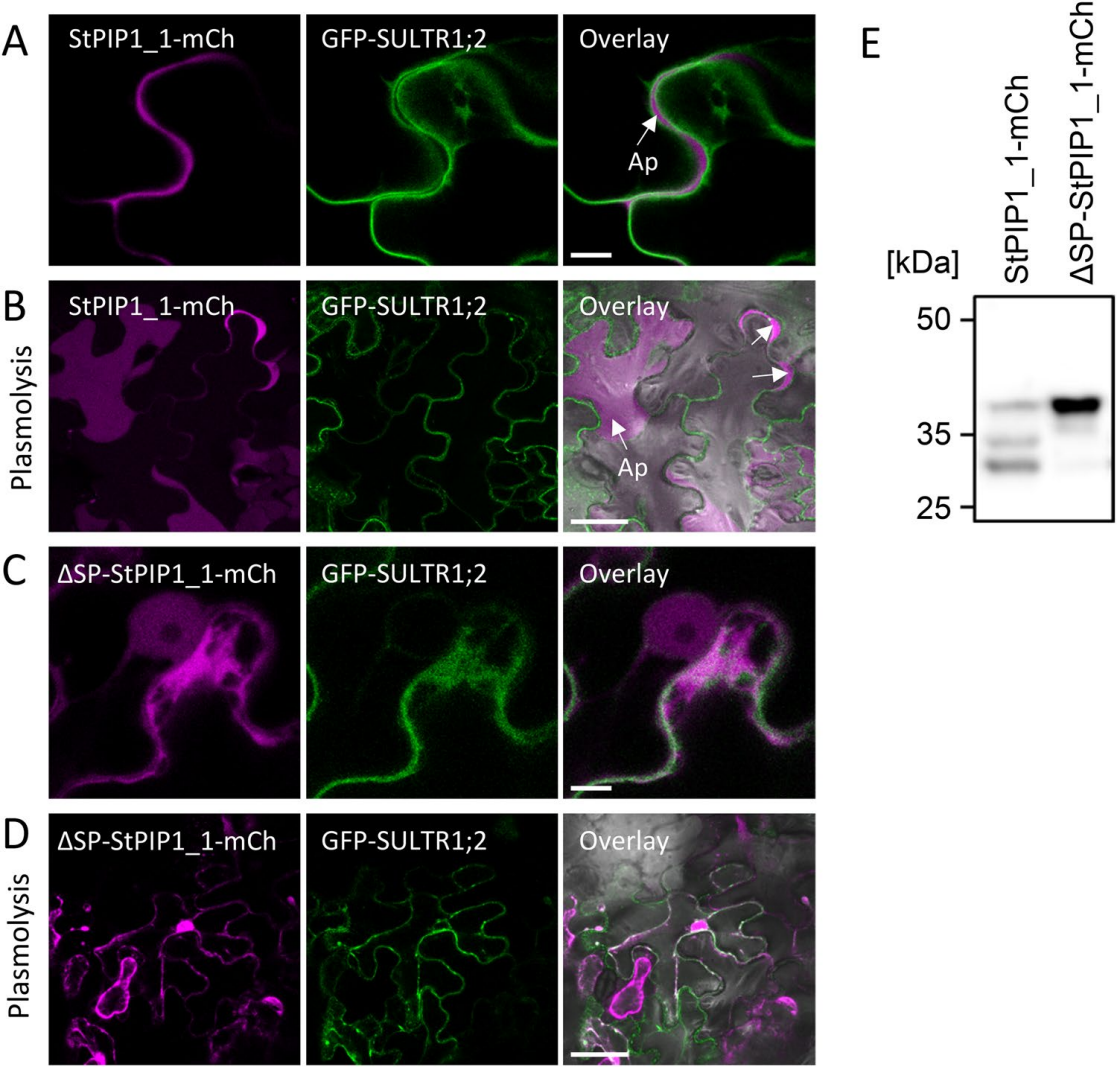
StPIP1_1 is exported into the apoplast. The coding region of StPIP1_1, or the coding region without the predicted signal peptide (ΔSP_StPIP1_1) fused to intron-containing mCherry (StPIP1_1-mCh and ΔSP_StPIP1_1-mCh) under the control of the 35S promoter, were transiently expressed in *N. benthamiana*, together with the GFP-tagged plasmamembrane protein SULTR1;2. (**A**) GFP, mCherry and merged fluorescence of SULTR1;2-GFP and StPIP1_1-mCh. Scale bars represent 10 µm. (**B**) Plasmolysis of *N. benthamiana* leaves after transient expression of StPIP1_1-mCh and SULTR1;2-GFP was induced by 150 mM NaCl. GFP, mCherry and merged fluorescence is shown. Scale bars represent 50 µm. (**C**) GFP, mCherry and merged fluorescence of SULTR1;2-GFP and ΔSP_StPIP1_1-mCh (without signal peptide). Scale bars represent 10 µm. (**D**) Plasmolysis in *N. benthamiana* leaves after transient expression of ΔSP_StPIP1_1-mCh and SULTR1;2-GFP was induced by 150 mM NaCl. GFP, mCherry and merged fluorescence is shown. Scale bars represent 50 µm. (**E**) Transient expression of fusion proteins in *N. benthamiana*. Proteins were extracted from *N. benthamiana* leaves expressing StPIP1_1-mCh or ΔSP_StPIP1_1-mCh 3 days after *Agrobacterium* infiltration and subjected to Western blot analyses using an α-RFP antibody.

StPIP1_1 and StPIP1_2 contain predicted signal peptides at their N-terminus suggesting that the proteins are targeted to the secretory pathway for apoplastic delivery. The localization of StPIP1_1 was determined by transient expression of a C-terminally mCherry tagged protein expressed under control of the CaMV 35S promoter in *Nicotiana benthamiana* (StPIP1_1-mCh). The coding region of StPIP1_1 was cloned in front of an intron-containing mCherry gene to ensure that mCherry signals are derived from plant-expressed transcripts. To address the importance of the signal peptide, we included a variant lacking the first 87 nucleotides, encoding the predicted signal peptide of 29 amino acids (ΔSP_StPIP1_1-mCh). As plasma membrane marker, Arabidopsis sulfate transporter SULTR1;2-GFP was co-expressed^33^. Confocal microscopy imaging revealed localization of StPIP1_1-mCherry fluorescence in the apoplastic space that lies between GFP-SULTR1;2-GFP labeled plasma membranes (Fig. 3A). Apoplastic localization was validated in cells plasmolyzed upon treatment with 150 mM NaCl (Fig. 3B), which confirmed StPIP1_1-mCherry accumulation in the extracellular space between cell walls and the plasma membrane. The variant lacking the predicted signal peptide, ΔSP_StPIP1_1-mCh displayed cytosolic and nuclear localization (Fig. 3C,D). Western blot analysis demonstrated expression of full-length StPIP1_1-mCh and ΔSP-StPIP1_1-mCh (Fig. 3E, Fig. S3). In addition, bands of smaller molecular weight were detected in samples from cells expressing StPIP1_1-mCh. The smaller variants may result from extracellular proteolytic cleavage of StPIP1_1-mCh or may represent partially or fully processed StPIP1_1 pre-pro-protein or the active StPIP1_1 peptide. Our analysis thus demonstrates that StPIP1_1 contains a functional signal peptide and is secreted to the apoplast via the secretory way where it is predicted to function as PAMP-induced peptide during plant defense responses.

### StPIP1_1 induces an oxidative burst in a SERK3A/B-dependent manner

**Figure 4:**
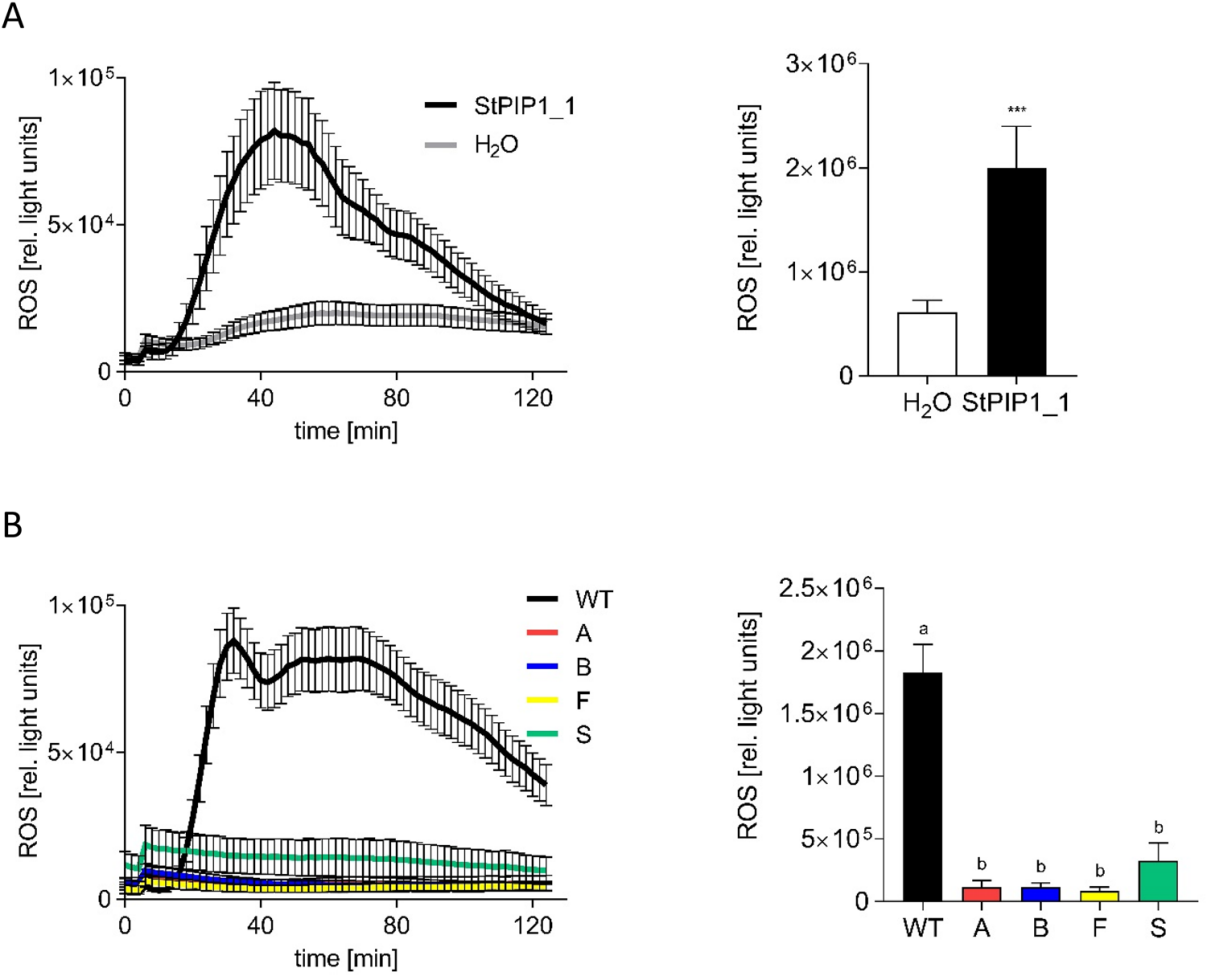
StPIP1_1 induces an oxidative burst in potato leaf disks in a StSERK3A/B-dependent manner. Leaf disks of phytochamber-grown potato plants were incubated with 100 nM StPIP1_1 and assayed for luminol-based ROS production. (**A**) ROS formation in leaf disks of wild type plants in response to StPIP1_1 (black) or water (grey). To quantify the ROS burst, light units up to 80 min were added (right panel). Error bars represent SEM. Data are derived from two independent experiments (n ≥ 24). Statistical analyses were performed using Mann Whitney U test (***p ≤ 0.01). (**B**) ROS formation in leaf disks of wild type (WT, black) and four independent *StSERK3A/B*-plants (A: red, B: blue, F: yellow, S: green) in response to StPIP1_1. To quantify the ROS burst, light units up to 80 min were added (right panel). Error bars represent SEM. Data are derived from two independent experiments (WT: n = 40, A,B,F,S: n = 24). Statistical analyses were performed using One-way-Anova. Different letters indicate significant differences.

One of the early responses to pathogen attack or PAMP/DAMP treatment is the oxidative burst. The oligopeptide StPIP1_1 was analyzed for its ability to induce the generation of ROS when added to leaf disks of potato plants. In contrast to water treatment, incubation with StPIP1_1 induced significantly enhanced levels of ROS (Fig. 4A). Since the leucine-rich receptor-like kinase BAK1 is part of the receptor complex required for perception of several peptides in Arabidopsis^34^, we addressed the importance of the co-receptor BAK1 for StPIP1_1-induced ROS formation. The oligopeptide was added to leaf disks of transgenic plants expressing an RNA interference construct against the two genes in potato, which encode the BAK1 isoforms *StSERK3A* and *StSERK3B*^27^. In these plants, StPIP1_1 was unable to induce an oxidative burst (Fig. 4B), suggesting a requirement for BAK1 as a co-receptor for StPIP1_1 perception.

### StPIP1_1 induces *FRK* gene expression in Arabidopsis in an RLK7-dependent manner

**Figure 5:**
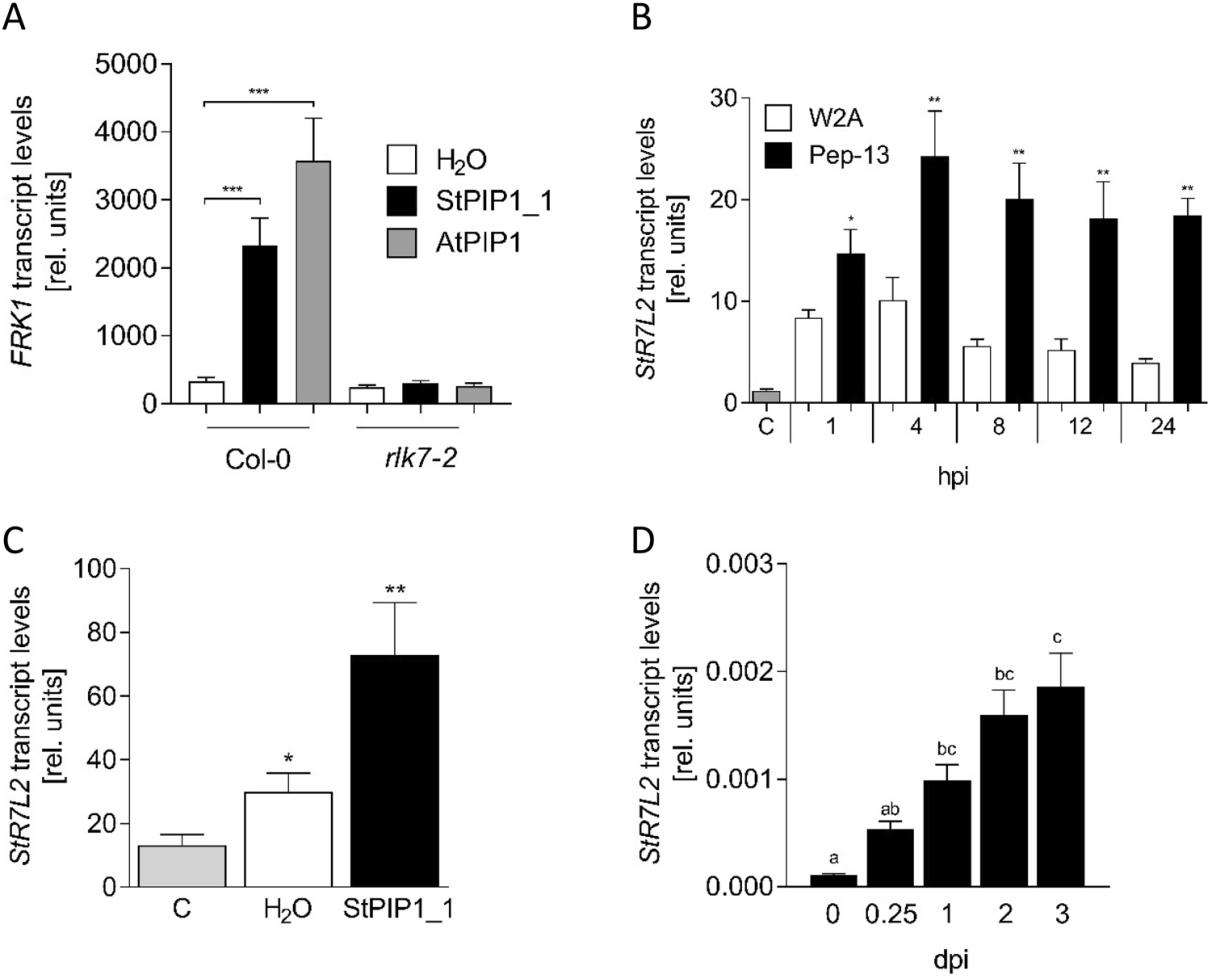
RLK7-dependent StPIP1_1-induced gene expression in Arabidopsis and expression of a potato gene encoding an LRR-RLK with sequence homology to RLK7. (**A**) StPIP1_1 from *S. tuberosum* induces *FRK1* transcript accumulation in a RLK7-dependent manner. Arabidopsis seedlings (Col-0 and the T-DNA insertion mutant rlk7-2 (SALK_083114) were treated with water (C, white bar), 1 µM StPIP1_1 (black bars) or AtPIP1 (grey bars). RNA was isolated after 4 h and subjected to qRT-PCR. Data are derived from three independent experiments (n = 7). Error bars represent SEM. Statistical analyses were performed using One-way-Anova. Different letters indicate statistically different values. (**B**) PAMP-induced expression of the LRR-RLK gene *StR7L2* with sequence homology to RLK7. RNA was isolated from untreated (C, grey bar), W2A (white bars) and Pep-13 (black bars) infiltrated potato leaves and subjected to qRT-PCR analyses. Expression was normalized to that of *StEF1α*. Data are derived from three independent experiments (n ≥ 5). Error bars represent SEM. Statistical analyses were performed using Mann Whitney U test (*p ≤ 0.05, **p ≤ 0.001) and show differences between W2A- and Pep-13-infiltrated plants. (**C**) StPIP1_1-induced expression of *StR7L2.* RNA was isolated from untreated leaves (“C”, grey bar) or 4 h after infiltration of water (white bars) or StPIP1_1- (black bars) and subjected to qRT-PCR analyses. Data shown were normalized to the expression of *StEF1α* and are derived from two independent experiments (n ≥ 11). Error bars represent SEM. Statistical differences between control plants and water-treated or StPIP1_1-treated plants were determined by Mann Whitney U test (*p ≤ 0.05; **p ≤ 0.001). (**D**) *P. infestans*-induced expression of *StR7L2*. RNA was isolated from *P. infestans*-infected leaves at the time points indicated and subjected to qRT-PCR analyses. Data shown were normalized to the expression of *StEF1α* and are derived from two independent experiments (n = 8). Error bars represent SEM. Different letters represent significant differences as determined by One-way-Anova.

Arabidopsis PIP1 has been described to bind to the receptor-like kinase (RLK) 7 and to activate defense responses^8^. To address whether StPIP1_1 is able to act in a similar manner, seedlings of the Arabidopsis wild type Col-0 and of the *rlk7-2* knock out mutant were treated with the oligopeptide StPIP1_1. As a control, AtPIP1 was included and the two peptides were analyzed for their ability to induce the expression of *FLG22-INDUCED RECEPTOR-LIKE KINASE 1* (*FRK1*). Consistent with published data, transcripts of *FRK1* accumulated in response to AtPIP1^8^. Interestingly, application of StPIP1_1 induced *FRK1* expression to a similar level (Fig. 5A). In the *rlk7-2* mutant, *FRK1* expression was not induced by neither AtPIP1 nor StPIP1_1, indicating that StPIP1_1 acts as a functional homolog of AtPIP1. This observation moreover suggests that StPIP1_1 might bind to an RLK7 homolog from potato. Comparing the amino acid sequence of RLK7 with the potato database, two putative RLKs were identified with 62 and 56% identity to RLK7 from Arabidopsis. Transcripts of RLK7-like 1 (StR7L1; PGSC0003DMG400004966) did not accumulate in response to Pep-13 treatment in infiltrated potato leaves, while RLK7-like 2 (StR7L2; PGSC0003DMG400005103) transcript levels were elevated (Fig. S4). To verify this expression, independent experiments were carried out. Pep-13 induced transcript accumulation of StR7L2 already 1 h after infiltration (Fig. 5B), and in response to StPIP1_1, significantly higher levels compared to untreated plants were observed after 4 h (Fig. 5C). Moreover, *P. infestans* infection led to enhanced levels of StR7L2 transcripts starting 1 day after inoculation, remaining high until the end of the experiment at 3 days (Fig. 5D). Thus, both *StPIP1_1* and StR7L2 were responsive to the PAMP Pep-13, the phytocytokine StPIP1_1 and to infection by *P. infestans*.

### StPIP1_1 and Pep-13 are rapidly degraded upon infiltration into potato leaves

**Figure 6:**
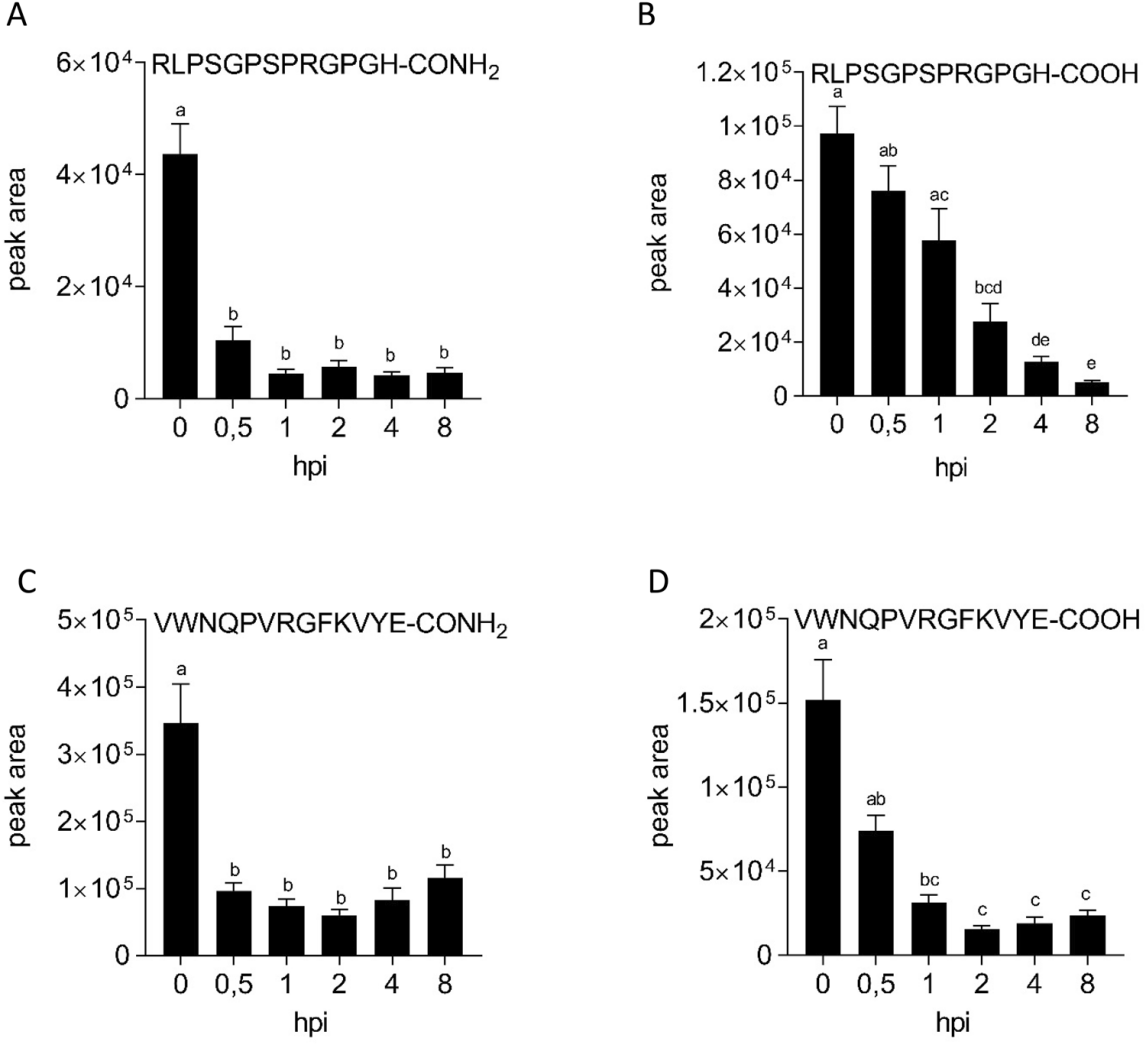
Peptides are rapidly degraded after infiltration into potato leaves. Phytochamber-grown potato plants were infiltrated with 100 µM amidated StPIP1_1 (**A**,**B**) or Pep-13 (**C**,**D**). Leaf samples were taken at the time points indicated and subjected to LC–MS analyses to determine the levels of amidated (**A**,**C**) and deaminated (**B**,**D**) peptides. Data are derived from two independent experiments (n = 16, except time point 0 in C and D: n = 12). Error bars represent SEM. Statistical analyses were performed using One-way-Anova. Different letters represent significant differences.

Exogenous application of peptides is routinely used to address their ability to activate immune responses, however, little is known about stability of peptides in plants. Our metabolomics analyses allowed the detection of exogenously applied peptides and their degradation products. We therefore screened the metabolomics data for StPIP1_1 peptides. When applied as an amide, StPIP1_1 was rapidly degraded to 20 and 10% within 30 min and 1 h, respectively (Fig. 6A). Interestingly, the deaminated acidic form of StPIP1_1 was detected within minutes. Two hours after infiltration, 30% of the initial amount was still present, decreasing to 13 and 5% after 4 and 8 h, respectively (Fig. 6B). This suggests that, upon infiltration, the amidated peptide was rapidly and efficiently converted to the acidic form, which, in turn, was degraded within 4 h. To analyze whether this effect is specific for StPIP1_1, we performed an equivalent experiment with Pep-13, the PAMP from *Phytophthora* species. As StPIP1_1, the amidated peptide was converted to the acidic form within 30 min (Fig. 6C), which was also degraded within 1 h (Fig. 6D). These results indicate that exogenously applied peptides do not persist in the apoplast, but are rapidly metabolized.

Our study shows that the potato homolog of AtPIP1 is secreted to the apoplast in a signal peptide-dependent manner, that the induction of early defense responses is dependent on BAK1 and that StPIP1_1 activates immune responses, including its own expression.

## Discussion

### StPIP1_1 is a functional homolog of AtPIP1

The potato genome contains genes encoding small secreted proteins which are induced by PAMP treatment or pathogen infection. Combest et al.^23^ reported that transcripts encoding the peptides StPIP1 to StPIP4 accumulate in response to infection with potato virus Y. These peptides show highest sequence homology to the Arabidopsis peptide AtPIP2^23^ (Fig. 1), which contains two GPS motifs. In contrast, the potato PIPs described here, StPIP1_1 and StPIP1_2, are more similar to AtPIP1 and only harbor a C-terminal SGPS motif. Combest et al.^23^ used a peptide of 23 amino acids covering the C-terminal GPS motif of StPIP1 (TLGGIKAGPSPGEGHKVVNSHHQ) to analyze immune responses. This peptide has highest sequence homology to the first motif in AtPIP2 (15 identical amino acids out of 23), and less to the C-terminal motifs in AtPIP2 (9 out of 23) or AtPIP1 (7 out of 23). Hou et al.^8^ used a peptide comprising the last 13 amino acids of AtPIP1. StPIP1_1 and StPIP1_2 have 11 and 10 identical amino acids, respectively, out of these 13 amino acids comprising the SGPS motif of AtPIP1. Moreover, AtPIP1, AtPIP2 and the two PIPs identified in our study represent the C-terminal amino acids, whereas StPIP1-4 have additional amino acids downstream of the GPS motif. Thus, we conclude that StPIP1_1 identified in this study is the potato PIP family member with the highest amino acid sequence homology to AtPIP1.

Similar to AtPIP1, exogenous application of a synthetic peptide covering the C-terminal 13 amino acids of StPIP1_1 to Arabidopsis seedlings induces FRK1 expression. Importantly, this induction does not take place in a mutant defective in *RLK7*, encoding the receptor-like kinase that binds AtPIP1^8^, suggesting that StPIP1_1 is a functional homolog of AtPIP1.

### StPIP1_1 induces immune responses in leaves

In response to treatment with StPIP1_1, we observed significant ROS production in potato leaf disks. In contrast, Combest et al.^23^ reported a ROS burst induced by StPIP1 only in nodes, but not in leaves. On the other hand, both StPIP1 and StPIP1_1 induced defense gene expression. In addition, we showed that defense metabolites, such as *p*-coumaroyl- and feruloyloctopamine, as well as *p*-coumaroyltyramine, accumulated in StPIP1_1-infiltrated plants. In potato, these compounds are synthesized in response to pathogen infection or PAMP treatment^32^. They are incorporated into the cell wall and are therefore postulated to contribute to pathogen resistance^31^. Indeed, overexpression of the tomato gene encoding the biosynthetic enzyme THT in transgenic plants resulted in enhanced levels of HCAAs and correlated with reduced growth of *Pseudomonas syringae* pv. *tomato*^35^.

The StPIP1_1-induced production of ROS and the accumulation of defense transcripts and metabolites in potato leaves suggest that StPIP1_1 indeed acts as a phytocytokine. In transgenic plants overexpressing StPIP1, no difference in gene expression to wild type plants was observed^23^. However, inoculation with potato virus Y resulted in the activation of a large number of genes in StPIP1 overexpressing plants, compared to the lower number of genes induced by PVY in control plants^23^, suggesting that StPIP1, once induced, amplifies the defense response. This is in accordance with Arabidopsis plants expressing AtPIP1, which displayed enhanced resistance to the root pathogen *Fusarium oxysporum*^8^.

### A signal peptide is required for apoplastic localization of StPIP1_1

In order to act as a phytocytokine, peptides have to be present in the apoplast. This can be achieved by secretion or by release into the apoplast after cellular damage. The proprotein of Arabidopsis AtPEP1 is released upon wounding and subsequently processed by Ca^2+^-dependent metacaspases^36^. More specifically, the metacaspase MC4 cleaves the precursor of PEP1 (PROPEP1) after a conserved arginine, resulting in the release of the C-terminal 23 amino acids that make up PEP1^36, 37^. In contrast, AtPIP1-GFP fusion proteins were present in the apoplast of non-disrupted cells after transient expression in *N. benthamiana* leaves^8^. Here we show that the apoplastic localization of potato StPIP1_1 is dependent on the presence of the predicted N-terminal signal peptide, suggesting that potato StPIP is targeted to the extracellular compartment by the secretory pathway. Similarly, IDA, a peptide with sequence similarity to PIPs, was also shown to be secreted in a signal peptide-dependent manner^38^.

*Nicotiana benthamiana* cells transiently expressing StPIP1_1-mCh contain proteins with a lower molecular weight than those expressing ΔSP_StPIP1_1-mCh, suggesting extracellular processing of the proprotein in the apoplast by yet unidentified proteases. In vitro experiments with GST-tagged AtPIP1 and AtPIP2, incubated with Arabidopsis protein extracts, also revealed processing of the proproteins, however, the proteases responsible or the nature of the released peptides had not been confirmed by proteomics^8^. For only a few apoplastic peptides, processing by proteases has been shown. The IDA peptide, for example, is released by subtilisin-like proteases^39^, whereas the apoplastic immune signaling peptide ZIP1 from maize is released from its proprotein by the action of Papain-like cysteine proteases^40^. In this context it is interesting to note that exogenously applied synthetic peptides are rapidly degraded, possibly by apoplastic proteases that are part of the immune barrier at the plant pathogen interface.

### StPIP1_1 perception requires the co-receptor BAK1

ROS production induced in potato leaf disks by StPIP1_1 was dependent on the functional co-receptors StSERK3A/B, the potato homologs of BAK1 from Arabidopsis, since no hydrogen peroxide formation was detected in *StSERK3A*/*B* silenced plants. Loss of ROS production in *StSERK3A*/*B*-silenced plants had also been observed in response to Pep-13 and flg22, but not chitin, suggesting that *StSERK3A*/*B* are required for peptide perception^27^. In Arabidopsis, the ROS burst induced by AtPIP1 was shown to be partially dependent on BAK1^8^. In addition, the induction of stomatal closure by AtPIP1 in Arabidopsis is also dependent on functional BAK1^41^. Thus, PIP peptides are suggested to be perceived by a receptor complex involving BAK1 in both Arabidopsis and potato. Data obtained in Arabidopsis moreover imply that such a receptor complex would include receptor kinase RLK7^(8, 22, 41^). Since StPIP1_1 is a functional homolog of AtPIP1, we searched for a possible homolog of RLK7 in potato. Comparison of the amino acid sequence of RLK7 to the potato database revealed homology to StR7L1 and StR7L2. As reported by Combest et al.^23^, transcripts of StR7L1 did not accumulate in virus-inoculated StPIP1 overexpressing plants, which is in accordance with our observation that expression is not induced after Pep-13 treatment. In contrast, transcripts of StR7L2 accumulate in response to PAMP treatment and pathogen infection. Thus, the encoded RLK is a candidate for the RLK7 homolog of potato.

Our data suggest that the potato homolog of AtPIP1, StPIP1_1, is secreted to the apoplast, causes an oxidative burst in a BAK1-dependent manner and induces defense gene expression as well as defense metabolite accumulation.

## Methods

### Plant culture and treatments

Potato plants (*Solanum tuberosum* cv. Désirée) were cultivated in sterile tissue culture in a phytochamber (16 h light, ~ 140 µE, 22 °C). Plants were transferred to steam-sterilized soil and grown for 4 weeks under long day conditions in a phytochamber (16 h light, ~ 140 µE, 60% humidity, 20 °C). PAMP treatment was performed with leaf disks that were cut out from 4-week-old potato plants with a biopsy puncher (4 mm diameter) and placed with the abaxial side onto the surface of 250 µl of water in a 96-well plate. The plate was incubated overnight at 22 °C in the dark. Water from the wells was removed and 100 µl sterilized fresh water per well was added. The plate was incubated for 30 min in the phytochamber (20 °C). Elicitation was performed by adding 100 nM StPIP1_1 (RLPSGPSPRGPGH), Pep-13 (VWNQPVRGFKVYE) or the nearly inactive analog W2A (VANQPVRGFKVYE^24, 27^). For whole plant assays, peptides were infiltrated as a 100 µM solution into the abaxial side of leaves of 3-week-old potato plants growing in a phytochamber. *Phytophthora infestans* infections were achieved by drop-inoculation of 10 µl of a zoospore suspension (1 × 10^5^ zoospores/ml) onto the abaxial leaf surface. Inoculated leaves were kept at 100% humidity for the duration of the experiment. As controls, water was pipetted onto the leaves^42^. *Nicotiana benthamiana* was grown from seeds in the greenhouse. Transient expression was performed with *Agrobacterium tumefaciens* containing binary vectors. Eleven-day-old Arabidopsis seedlings (Col-0 and *rlk7-2* (SALK_083114)) grown in microtiter plates were treated with 1µM StPIP1_1.

### RNA expression analyses

RNA was isolated from potato leaves, leaf disks or Arabidopsis seedlings as described^26^. DNase digestion (RNase-free DNase Set, Qiagen) and cDNA synthesis using Maxima H Minus First Strand cDNA Synthesis Kit (Thermo Fisher Scientific) were performed according to the manufacturer´s instructions. For quantitative PCR, Maxima Probe qPCR MasterMix (Thermo Fischer Scientific) was used and the samples were run on an Mx3005P qPCR system (Agilent).

The following primers and real time probes were used: *StPIP1_1*: 5′-GGAAATTCGTCTCGCAACTT-3′, 5′-CTGAAGGTAGCCTCTCAAGCA-3′ and Roche Universal Probe Library Probe #155, *StPR1*: 5′-TCAGTTCGACTAGGTTGTGGTC-3′, 5′-GTCCGACCCAGTTTCCAAC-3′ and probe #157, *St4CL*: 5′-TGCTGTTGTCCCAATGATAGA-3′, 5′-TGATCTAACAACAAAAGCCACTG-3′ and probe #7, *StTHT*: 5′-CCTCCTTAGAGGGCTTGCTT-3′, 5′-AGTACGGATGGCCCGTAGA-3′ and probe #144, *StRLK7*: 5′-TGGAAAGCCTAGAGAATGGTACA-3′, 5′-CAAATGAAACGAGCACCACA-3′ and probe #83, *StEF1α*: 5′-CACTGCCCAGGTCATCATC-3′, 5′-GTCGAGCACTGGTGCATATC-3′ and Roche Universal Probe Library Probe #163, *AtFRK1*: 5′-GAGACTATTTGGCAGGTAAAAGGT-3′, 5′-AGGAGGCTTACAACCATTGTG-3′ and probe #33.

### ROS assay

ROS analyses were performed as described^43^ with the following modifications: Each well contained 200 µl of water supplied with 5 µM luminol L-012 (Wako Chemicals), 2 µg horseradish peroxidase (Fluka) and 100 nM peptide.

### Liquid chromatography-mass spectrometry measurements

Leaf disks from 4-week-old infiltrated potato plants were cut out at defined time points after infiltration and frozen in liquid nitrogen. Chromatographic separations of methanolic extracts were performed as described previously^44, 45^ with the following modifications of the binary gradient with 0.150 µl/min: 0–1 min isocratic 95% A (water/formic acid, 99.9/0.1 [v/v]) and 5% B (acetonitrile/formic acid, 99.9/0.1 [v/v]); 1–10 min linear from 5 to 60% B; 10–10.2 min linear to 95% B; 10.2–12 min isocratic 95% B; 12–14 min isocratic 5% B. Eluting compounds were detected from m/z 50 to 1000. LC–MS profiling data were analysed as described previously^45, 46^.

Infiltrated peptides of StPIP1_1 and Pep-13, which both had a carboxy-terminal amidation, were annotated by comparison of consistent retention time, accurate mass and MS/MS identity to the applied authentic standards. Deamidated peptides and peptide fragments were annotated by accurate mass as calculated from possible peptide structures generated in silico and by MS/MS sequence interpretation.

### Subcellular localization of StPIP1_1-mCherry

A gene encompassing the coding region of StPIP1_1 fused to mCherry containing an intron was synthesized by GeneArt (Thermofisher). The gene was cloned into a binary vector under the control of the 35S promoter by Golden Gate Cloning^47^. As plasma membrane marker, Arabidopsis SULTR1;2 was included^33^. The coding sequence of SULTR1;2 without the stop codon was amplified from Arabidopsis cDNA using the primers 5′-GGGGACAAGTTTGTACAAAAAAGCAGGCTTCATGTCGTCAAGAGCTCACCCTGTGG-3′ and 5′-GGGGACCACTTTGTACAAGAAAGCTGGGTCTCAGACCTCGTTGGAGAGTTTTGG-3, cloned into pDONR221 via BP Ligation, and mobilized into pB7FWG2^48^ to generate *ProCaMV35:SULTR1;2-GFP*. *Agrobacterium tumefaciens* GV3101 containing *StPIP1_1-mCh*, *SULTR1;2* or the silencing suppressor *p19* in a 1:1:2 ratio were co-infiltrated for transient expression in *N. benthamiana*. Three days after infiltration, microscopy was performed using a Zeiss LSM 780 inverted microscope using a 40× water-immersion objective. The excitation wavelengths for GFP and mCherry were 488 nm and 561 nm, respectively, and emission was detected between 495 and 550 nm (GFP) and 570 and 620 nm (RFP). Bright field images were captured with the TPMT detector. Plasmolysis was induced by application of 150 mM NaCl to excised leaf discs mounted on object slides.

### Immunoblot analysis

Proteins were analyzed from *N. benthamiana* leaves expressing StPIP1_1-mCherry or ΔSP-StPIP1_1-mCherry 3 days after Agrobacterium infiltration. Leaves were ground in liquid nitrogen and the leaf material was mixed with 2× Laemmli buffer in a 1:1 ratio. For each sample, 20 µl were loaded on a 10% SDS gel and blotted for 1 h. Blots were blocked in 1× TBS with 2% [w/v] milk powder and 2% [w/v] BSA for 1 h at RT. For protein detection, blots were incubated over night at 4 °C with a primary mouse α-RFP antibody (1:1000; 6G6, Chromotek) and subsequently for 2 h at RT with a secondary α-mouse antibody coupled to horseradish peroxidase (1:20,000; A9044, Sigma-Aldrich). Western blot images were acquired with a FluorChem system detection using ECL-Prime (Amersham/Cytiva) for detection.

The authors confirm that all methods were carried out in accordance with the relevant guidelines.

### Supplementary Information

Supplementary Figures.

## Data Availability

The datasets used and/or analysed during the current study available from the corresponding author on request.
