# Supplementary material for: The secreted PAMP-induced peptide StPIP1_1 activates immune responses in potato
Source: Sci Rep. 2023 Nov 23;13:20534. doi: 10.1038/s41598-023-47648-x (PMC10667265; doi:10.1038/s41598-023-47648-x)
Supplement: Supplementary file 1 — Supplementary Figures. [file 41598_2023_47648_MOESM1_ESM.pptx]

## Slide 1
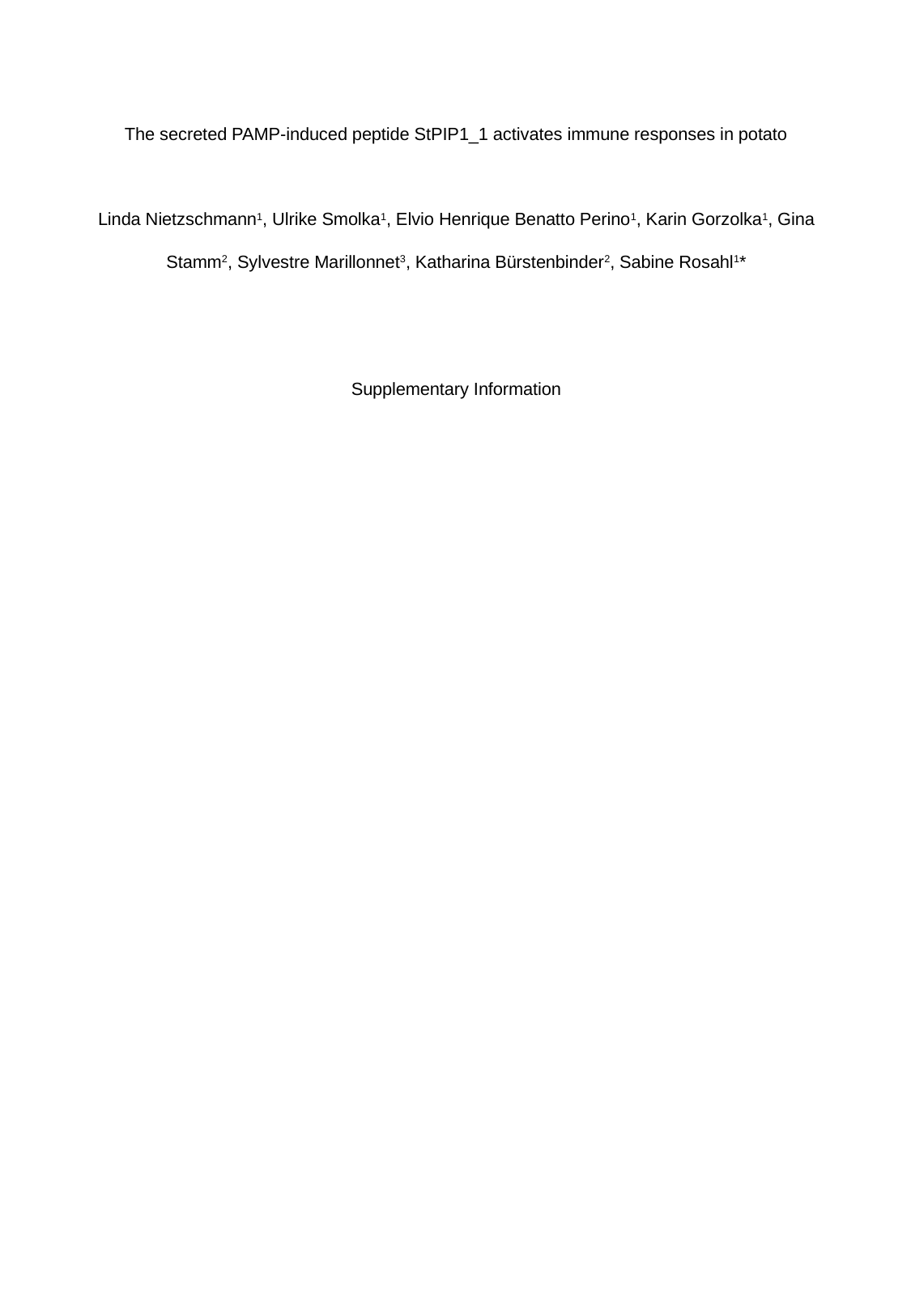

The secreted PAMP-induced peptide StPIP1_1 activates immune responses in potato
Linda Nietzschmann1, Ulrike Smolka1, Elvio Henrique Benatto Perino1, Karin Gorzolka1, Gina Stamm2, Sylvestre Marillonnet3, Katharina Bürstenbinder2, Sabine Rosahl1*
Supplementary Information

## Slide 2
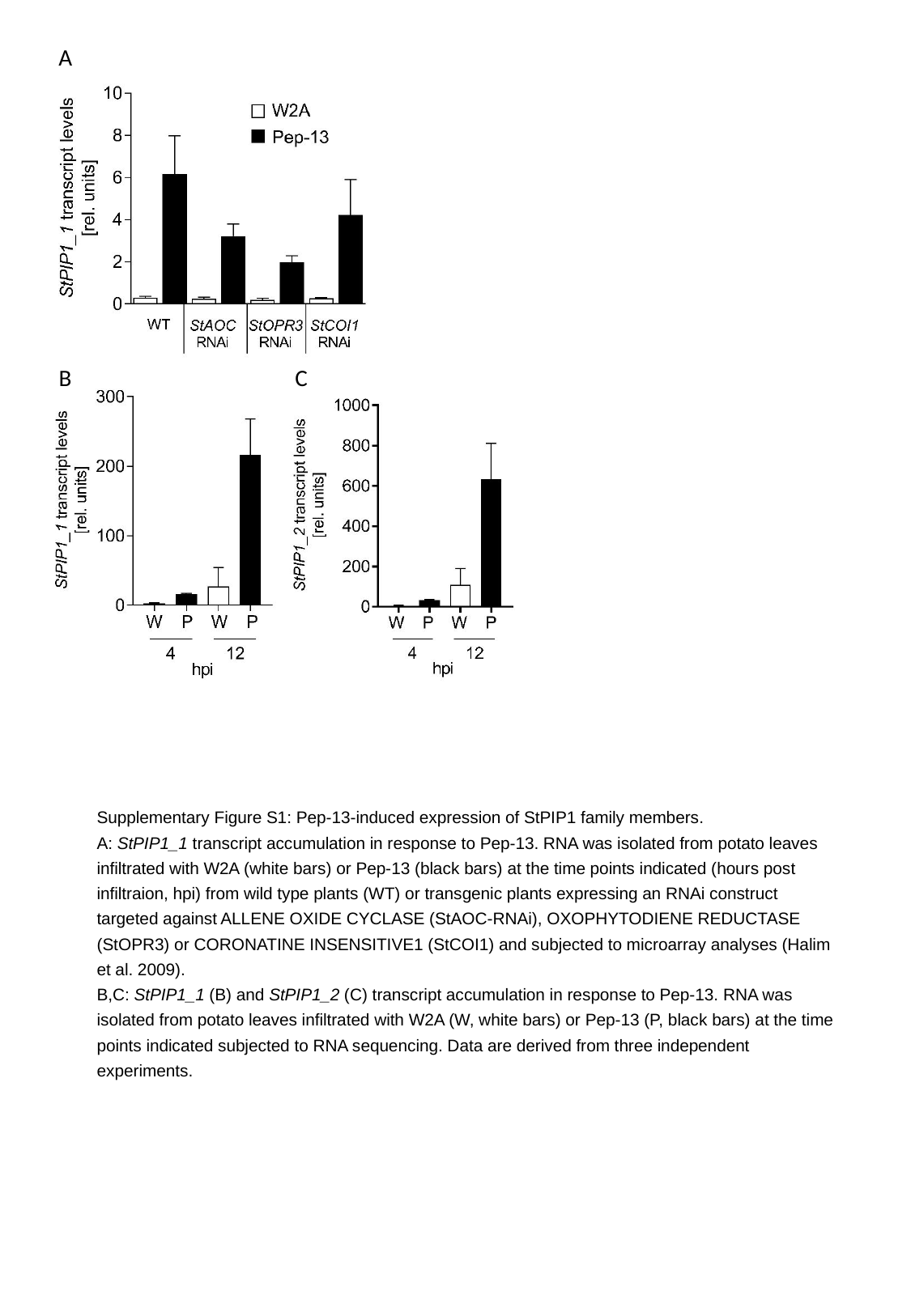

A
B
C
Supplementary Figure S1: Pep-13-induced expression of StPIP1 family members.
A: StPIP1_1 transcript accumulation in response to Pep-13. RNA was isolated from potato leaves infiltrated with W2A (white bars) or Pep-13 (black bars) at the time points indicated (hours post infiltraion, hpi) from wild type plants (WT) or transgenic plants expressing an RNAi construct targeted against ALLENE OXIDE CYCLASE (StAOC-RNAi), OXOPHYTODIENE REDUCTASE (StOPR3) or CORONATINE INSENSITIVE1 (StCOI1) and subjected to microarray analyses (Halim et al. 2009).
B,C: StPIP1_1 (B) and StPIP1_2 (C) transcript accumulation in response to Pep-13. RNA was isolated from potato leaves infiltrated with W2A (W, white bars) or Pep-13 (P, black bars) at the time points indicated subjected to RNA sequencing. Data are derived from three independent experiments.

## Slide 3
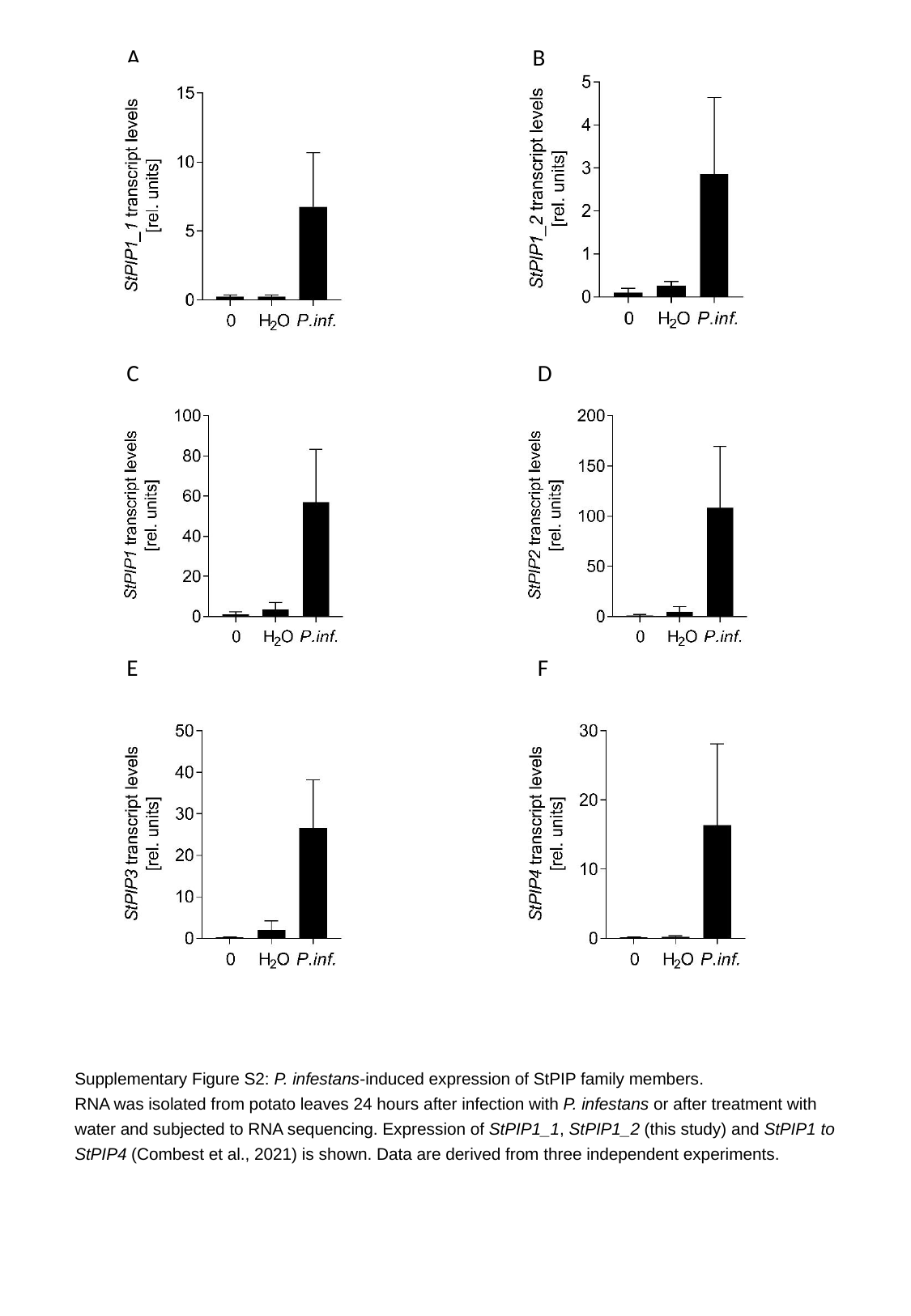

A
B
C
D
E
F
Supplementary Figure S2: P. infestans-induced expression of StPIP family members.
RNA was isolated from potato leaves 24 hours after infection with P. infestans or after treatment with water and subjected to RNA sequencing. Expression of StPIP1_1, StPIP1_2 (this study) and StPIP1 to StPIP4 (Combest et al., 2021) is shown. Data are derived from three independent experiments.

## Slide 4
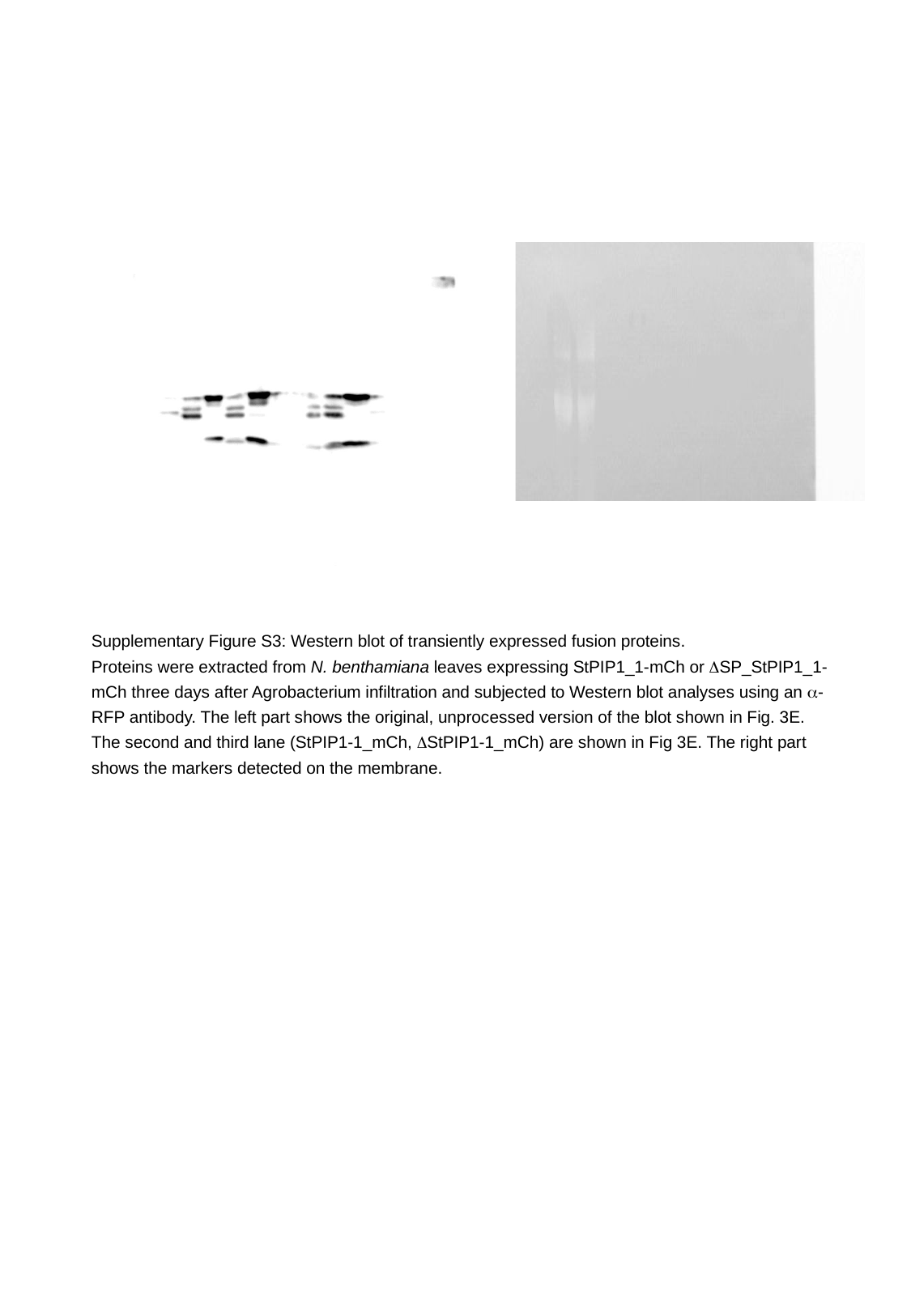

Supplementary Figure S3: Western blot of transiently expressed fusion proteins.
Proteins were extracted from N. benthamiana leaves expressing StPIP1_1-mCh or ΔSP_StPIP1_1-mCh three days after Agrobacterium infiltration and subjected to Western blot analyses using an α-RFP antibody. The left part shows the original, unprocessed version of the blot shown in Fig. 3E. The second and third lane (StPIP1-1_mCh, ΔStPIP1-1_mCh) are shown in Fig 3E. The right part shows the markers detected on the membrane.

## Slide 5
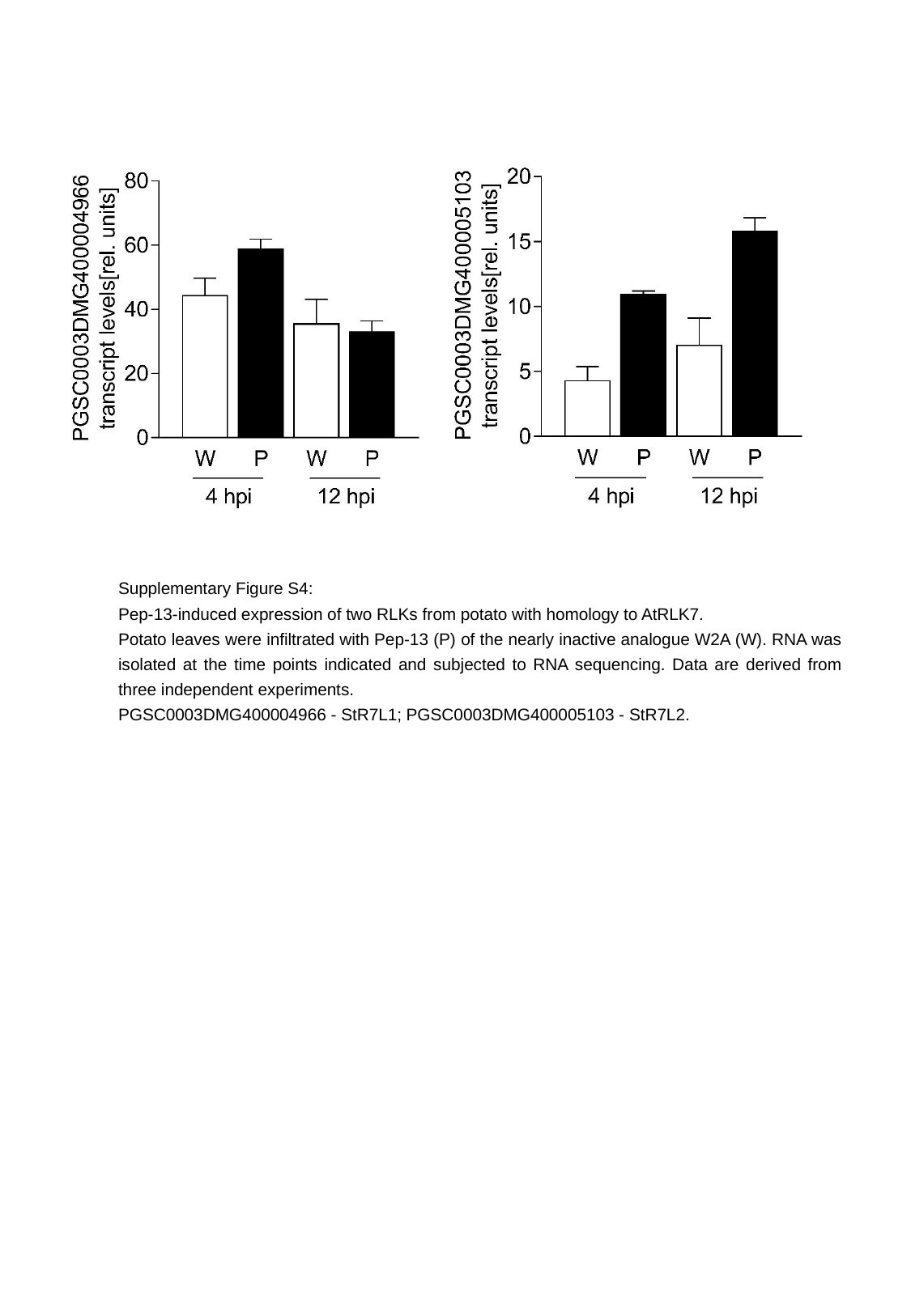

Supplementary Figure S4:
Pep-13-induced expression of two RLKs from potato with homology to AtRLK7.
Potato leaves were infiltrated with Pep-13 (P) of the nearly inactive analogue W2A (W). RNA was isolated at the time points indicated and subjected to RNA sequencing. Data are derived from three independent experiments.
PGSC0003DMG400004966 - StR7L1; PGSC0003DMG400005103 - StR7L2.
